# Efficacy and safety of copanlisib in relapsed/refractory B-cell non-Hodgkin lymphoma: A meta-analysis of prospective clinical trials

**DOI:** 10.3389/fimmu.2022.1034253

**Published:** 2022-11-11

**Authors:** Jinjin Wang, Hui Zhou, Mingchun Mu, Ailin Zhao, Zhaolun Cai, Linfeng Li, Mengyao Wang, Ting Niu

**Affiliations:** ^1^ Department of Hematology, West China Hospital, Sichuan University, Chengdu, Sichuan, China; ^2^ Gastric Cancer Center, West China Hospital, Sichuan University, Chengdu, Sichuan, China

**Keywords:** copanlisib, rituximab, R/R B-NHL, efficacy, safety, meta-analysis

## Abstract

**Background:**

Copanlisib is an intravenously administered pan-class I PI3K inhibitor that has been demonstrated to have appreciable effects in the treatment of patients with lymphoma. The purpose of this meta-analysis was to evaluate the efficacy and safety of copanlisib for treating patients with relapsed/refractory (R/R) B-cell non-Hodgkin lymphoma (B-NHL).

**Methods:**

PubMed, Web of Science, EMBASE, and the Cochrane Central Register of Controlled Trials were searched for relevant studies published prior to July 2022. The efficacy evaluation included complete response rate (CR), partial response rate (PR), rate of stable disease (SDR), overall response rate (ORR), disease control rate (DCR), rate of progressive disease (PDR), median progression-free survival (PFS), and median overall survival (OS). Any grade adverse events (AEs) and grade ≥3 AEs were synthesized to assess its safety.

**Results:**

Eight studies with a total of 652 patients with R/R B-NHL were identified. The pooled CR, PR, ORR, SDR, DCR, and PDR from all 8 articles were 13%, 40%, 57%, 19%, 86%, and 9%, respectively. The CR and ORR of combination therapy with rituximab were higher than those with copanlisib monotherapy for R/R B-NHL (34% vs. 6%, p<0.01; 89% vs. 42%, p<0.01). For patients with R/R indolent B-NHL, CR and ORR were lower with copanlisib monotherapy than with combination therapy with rituximab (7% vs. 34%, p<0.01; 58% vs. 92%, p<0.01). In R/R B-NHL patients receiving copanlisib monotherapy and combination therapy with rituximab, the risk of any grade AEs was 99% and 96%, respectively, and the risk of grade ≥3 AEs was 84% and 91%, respectively. The common any grade AEs included hyperglycemia (66.75%), hypertension (48.57%), diarrhea (35.06%), nausea (34.98%) and fatigue (30.33%). The common grade ≥3 AEs included hyperglycemia (45.14%), hypertension (35.07%), and neutropenia (14.75%). The comparison of AEs between the copanlisib monotherapy and the combination therapy with rituximab showed that hyperglycemia of any grade (p<0.0001), hypertension of any grade (p=0.0368), fatigue of any grade (p<0.0001), grade ≥3 hypertension (p<0.0001) and grade ≥3 hyperglycemia (p=0.0074) were significantly different between the two groups.

**Conclusion:**

Our meta-analysis demonstrated that the efficacy of both copanlisib monotherapy and combination therapy with rituximab in patients with R/R B-NHL was satisfactory, while treatment-related AEs were tolerable. Compared with copanlisib monotherapy, combination therapy with rituximab showed superior efficacy for treating R/R B-NHL, and its safety was manageable.

**Systematic Review Registration:**

https://inplasy.com/inplasy-2022-10-0008/, identifier INPLASY2022100008.

## Introduction

B-cell non-Hodgkin lymphoma (B-NHL) is a large group of lymphomas that can be divided into indolent B-NHL and aggressive B-NHL. A survey found that the incidence of both indolent and aggressive B-NHLs has increased in recent years (1). Patients with indolent B-NHL are generally considered incurable, often recur repeatedly, receive multiple lines of antitumor therapy and are prone to drug resistance (2). Approximately 25%-30% of patients with aggressive B-NHL have a poor reaction to first-line therapy or relapse (3). Diffuse large B-cell lymphoma is a common aggressive B-NHL, accounting for approximately 30%-58% of NHL cases (4). A large-scale cohort study showed that 2778 patients with refractory diffuse large B-cell lymphoma had a median overall survival (OS) of 5.9 months and a 2‐year OS rate of 16% (5).

Treatment of B-NHL mainly includes alkylating agents, combination chemotherapy, chemoimmunotherapy, high-dose chemotherapy + autologous/allogeneic stem cell transplantation, radiotherapy, chimeric antigen receptor T-cell therapy, and so on (6, 7). However, less than half of patients with relapsed/refractory (R/R) indolent B-NHL were responsive to subsequent treatment (8, 9). No standard treatment has been developed for patients with R/R aggressive B-NHL. Currently, high-dose salvage chemotherapy or bone marrow transplantation are often used, but the overall effect is not satisfactory (10). The management of patients with R/R B-NHL has become a major difficulty for hematologists. Therefore, it is necessary to develop more effective drugs for treating patients with R/R B-NHL.

The B-cell receptor (BCR) signaling pathway accounts for much of the development of B-cell lymphoma. In the BCR pathway, phosphatidylinositol 3-kinase (PI3K) and Bruton tyrosine kinase play significant roles (11). PI3K is a key downstream effector of the BCR (12). Since PI3K is important in carcinogenesis, it has become one of the potential targets for lymphoma treatment. PI3K is classified into three types (I, II, III) based on their distinctive substrates and structures. Class I PI3K is a heterodimer formed from class IA and class IB PI3K, both of which consist of catalytic subunits (p110 or p110γ) and regulatory subunits (p85 or p101) (13). Class II PI3K comprises only one catalytic subunit, including three isoforms: PI3K–C2α, PI3K–C2β, and PI3K–C2γ (14). Class III PI3K is only formed from Vps34p. Of the three classes of PI3Ks, class I is most closely associated with tumorigenesis and progression (12, 15).

Class I PI3K includes four isoforms, namely, PI3Kα, PI3Kβ, PI3Kγ, and PI3Kδ. PI3kα and PI3Kβ generally exist in various kinds of cells, whereas PI3Kγ and PI3Kδ are mainly expressed in the hemopoietic system (16). PI3K inhibitors can be classified into pan-PI3K inhibitors, isoform-specific inhibitors, and dual PI3K/mTOR inhibitors according to their different selectivity. Pan-PI3K inhibitors are effective against all four isoforms of class I PI3K, such as buparlisib, which has not been approved for treating lymphoma by the Food and Drug Administration (FDA) (17). Isoform-specific inhibitors are selective for a specific isoform of PI3K, and they can be separated into selective PI3Kα inhibitors, selective PI3Kβ inhibitors, selective PI3Kγ inhibitors, and selective PI3Kδ inhibitors. Dual PI3K/mTOR inhibitors can also specifically bind to a domain of mTOR, so they can simultaneously inhibit mTOR. To date, dual PI3K/mTOR inhibitors have not been approved for cancer treatment, but clinical trials are underway (18). Currently, the only oral PI3K inhibitors approved by the FDA for the treatment of lymphoma are idelalisib (PI3Kδ inhibitor) and duvelisib (PI3Kδ and PI3Kγ inhibitor) (19, 20). However, these two oral drugs have serious safety problems in clinical application, such as severe intestinal adverse events (AEs) and infections (21). Therefore, the FDA also gives a corresponding warning statement in the drug label.

Copanlisib is a pan-class I PI3K inhibitor that is highly selective for PI3Kα and PI3Kδ isoforms (22). It is administered intravenously. The CHRONOS-1 (a large multicenter phase 2 clinical trial) study showed a considerable overall response rate (ORR, 60.6%), progression-free survival (PFS), and OS with copanlisib for R/R indolent lymphoma, as well as satisfactory safety (23). Based on the results of CHRONOS-1, the FDA rapidly approved copanlisib for treating relapsed follicular lymphoma in 2017. Most B-NHLs express the CD20 antigen, so rituximab is one of the standard options for treatment. However, due to the drug resistance of patients with R/R B-NHL and the poor efficacy of monotherapy, researchers have been exploring combination regimens. A randomized double-blind phase 3 trial (CHRONOS-3) indicated that the ORR (81% vs. 48%) and median PFS (21.5 months vs. 13.8 months) in the copanlisib plus rituximab group were significantly higher than those in the placebo plus rituximab group (24).

At present, various researchers have been exploring the curative effect of copanlisib-containing regimens in patients with B-NHL. Therefore, this meta-analysis was performed to comprehensively evaluate the efficacy and safety of monotherapy or combination therapy with rituximab for patients with R/R B-NHL to provide a basis for clinical practice.

## Methods

### Search strategy

Original studies that described the efficacy or safety of copanlisib monotherapy or combination therapy, including copanlisib plus rituximab, for treating B-NHL were systematically searched for in the PubMed, Web of Science, EMBASE, and Cochrane Central Register of Controlled Trials. The search terms were combined as follows: “Copanlisib OR Aliqopa OR BAY80-6946” AND “lymphoma”. The search included only articles published before July 2022 and had no language restrictions. This meta-analysis followed the Preferred Reporting Items for Systematic reviews and Meta-Analyses (PRISMA) guidelines.

### Inclusion and exclusion criteria

We followed the following inclusion criteria to screen the literature: 1) prospective clinical trials at any stage; 2) studies including patients diagnosed with R/R B-NHL; 3) articles studying copanlisib monotherapy or combination therapy with rituximab; and 4) clinical trials reporting any data involving their efficacy or safety.

The exclusion criteria were as follows: 1) no available data of efficacy or safety; 2) reviews, case reports, news, editorials, meta-analyses, and meeting/conference abstracts.

### Data extraction and quality assessment

Two authors (JW and HZ) independently screened the literature and collected the data, and any difference was settled by the third author. The extracted data were sorted into a designed spreadsheet that mainly included the first author, ClinicalTrials.gov number, phase, study design, number of patients, disease, ages, treatment, prior lines of anticancer therapy, any grade AEs, grade ≥3 AEs, complete response rate (CR), partial response rate (PR), rate of stable disease (SDR), ORR, disease control rate (DCR), rate of progressive disease (PDR), median PFS, and median OS. These terms are defined in the Supplementary material. For all enrolled studies, we only extracted information about copanlisib monotherapy or combination therapy with rituximab, for treating B-NHL. For the included randomized controlled trials (RCTs), the quality was estimated by the Cochrane Collaboration Risk of Bias Tool (25). The methodological index for nonrandomized studies (MINORS) was utilized to assess the quality of the non-RCTs (26).

### Statistical analysis

Statistical analysis of the data was performed using R 4.1.1 software. The I² statistic test was applied to appraise the heterogeneity among studies. The value of the I² statistic is 0 to 100%. I²<25% indicates mild heterogeneity, I² 25–50% means moderate heterogeneity, and I2>50% manifests obvious heterogeneity. A fixed-effects model was employed if the I^2^ statistic was low (I^2^ ≤ 50%), while a random-effects model was utilized with I^2^>50%. Subgroup analysis (copanlisib vs. including copanlisib plus rituximab; R/R indolent B-NHL vs. R/R aggressive B-NHL) was employed to address any heterogeneity.

## Results

### Study characteristics

**Figure 1:**
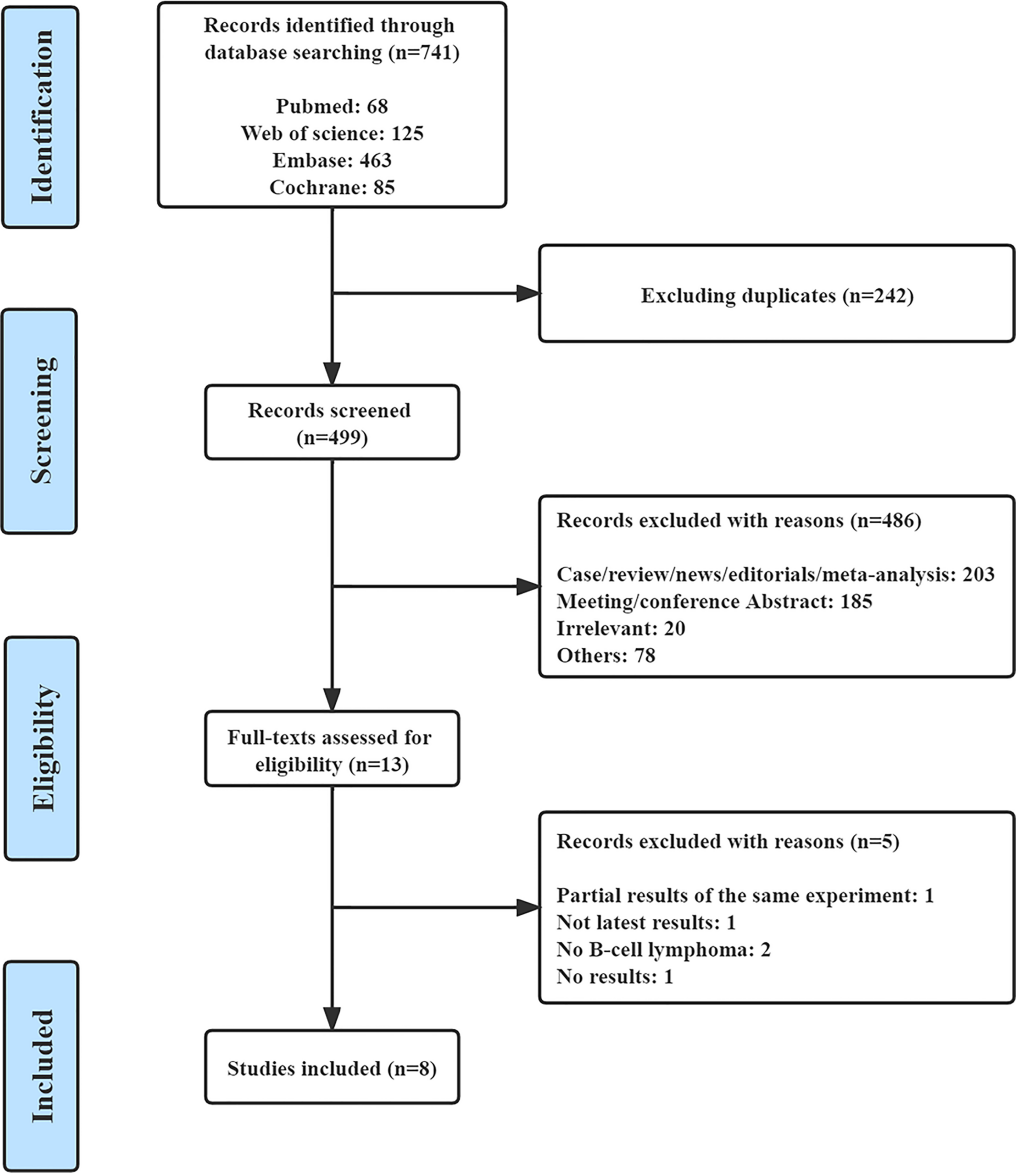
The flow chart.

A total of 741 records were retrieved from PubMed (n=68), Web of Science (n=125), EMBASE (n=463), and the Cochrane Central Register of Controlled Trials (n=85). After removing 242 duplicate studies and 486 articles for various reasons, we read the full text of 13 articles. Finally, eight qualified studies were included in the meta-analysis (23, 24, 27–32). **Figure 1** shows the complete screening process. All eight articles were prospective clinical trials, including three phase I trials, three phase II trials, and two phase III trials. The included studies were published from 2016 to 2022. The features of all of the eligible studies are shown in **Table 1**. Altogether, 652 patients with R/R B-NHL were included, of whom 516 had R/R indolent B-NHL, 127 had R/R aggressive B-NHL, and the remaining nine were unable to distinguish between indolent and aggressive B-NHL. The median age of all patients ranged from 60 to 72 years.

**Table 1:** The characteristics of included studies.

N0.	Study	Clinical Trials. gov number	Phase	Study design	Number of patients	Disease	Ages(years), medium(range)	Treatment	Prior lines of therapy	Any grade AEs (n)	grade ≥3 AEs (n)	CR (%)	PR (%)	ORR (%)	SDR (%)	DCR (%)	PDR (%)	Median PFS (m)	Median OS (m)
1	Liu et al., 2022 (27)	NCT03498430	I	single-arm	13	R/R indolent B-NHL	40 (30–64)	copanlisib	1-8	13	10	0	58.3%	58.30%	41.6%	100%	0	—	—
2	Lenz et al., 2020 (28)	NCT02391116	II	single-arm	67	R/R aggressive B-NHL	69(25–93)	copanlisib	1-13	65	58	7.5%	11.9%	19.40%	20.9%	40.30%	44.80%	1.8	7.4
3	Dreyling et al., 2017 (1) (29)	NCT01660451 -part A	II	single-arm	33	R/R indolent B-NHL	66.5 (22–90)	copanlisib	1-10	33		6.3%	34.4%	43.80%	45.5%	90.60%	3%	9.8	21.9
	Dreyling et al., 2017 (2) (29)				34	R/R aggressive B-NHL				—	—	0	23.5%	29.40%	17.6%	47.10%	32.40%	—	—
4	Dreyling et al., 2020 (23)	NCT01660451 -part B	II	single-arm	142	R/R indolent B-NHL	63 (25–82)	copanlisib	2-9	140	118	16.9%	43.7%	60.60%	28.9%	89.40%	2.10%	12.5	42.6
5	Patnaik et al., 2016 (30)	NCT00962611	I	dose-escalation	9	R/R B-NHL	72 (40–84)	copanlisib	1-8	8	6	11.1%	66.7%	77.80%	0	77.80%	22.20%	—	—
6	Morschhauser et al., 2020 (31)	NCT02155582	I	single-arm	26	R/R aggressive B-NHL	61 (38–80)	copanlisib	—	—	—	3.8%	19.2%	23.10%	—	—	—	—	—
7	Matasar et al., 2021a (24)	NCT02367040	III	double-blind, randomised	307	R/R indolent B-NHL	63 (54–70)	copanlisib+rituximab	—	293	280	33.9%	44.6%	81%	11.7%	89%	2%	21.5	—
8	Matasar et al., 2021b (1) (32)	NCT02626455	III	double-blind, randomised	10	R/R indolent B-NHL	62 (41-82)	copanlisib++R-B	1-3	10	7	50%	40%	90%	10%	100%	0	—	—
	Matasar et al., 2021b (2) (32)				11	R/R indolent B-NHL	64 (46-78)	copanlisib++R-CHOP		11	10	30%	70%	100%	0	100%	0	—	—

R/R, relapsed or refractory; B-NHL, B-cell non-Hodgkin lymphoma; R-B, rituximab + bendamustine; R-CHOP, rituximab + cyclophosphamide, doxorubicin, vincristine, and prednisone.

In the eight articles, patients received copanlisib monotherapy in six trials and combination therapy including copanlisib plus rituximab in the remaining 2 trials (copanlisib + rituximab, copanlisib + rituximab + bendamustine, copanlisib + rituximab + cyclophosphamide/doxorubicin/vincristine/prednisone). The participants had received 1 to 13 prior lines of anticancer therapy. Seven of the included studies reported complete information on efficacy (CR, PR, SDR, ORR, DCR, and PDR), and six trials showed full information on safety. **Supplementary Table S1** shows information on the dose of copanlisib, frequency of administration, median duration of treatment, follow-up time, and modification of doses (reduction or interruption, or delay) or discontinuation due to AEs in the included studies. The doses of copanlisib in all studies included 45 mg, 60 mg, 0.4 mg/kg, and 0.8 mg/kg, and the frequency of copanlisib intravenous infusion was Days 1, 8, and 15 (28 days per cycle). The median duration of treatment ranged from 6 to 33.2 weeks. The rates of discontinuation of treatment due to AEs ranged from 15.4% to 31.3%.

### Efficacy

**Figure 2:**
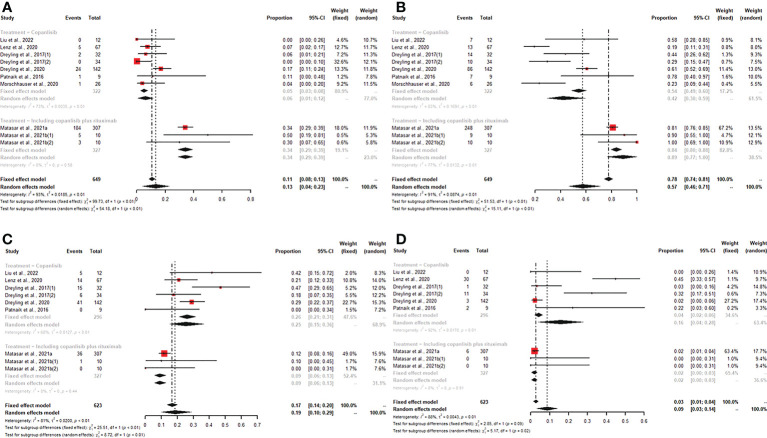
The pooled CR **(A)** and ORR **(B)** in patients with R/R B-NHL receiving copanlisib monotherapy were significantly higher than those receiving combination therapy, including copanlisib plus rituximab, while results for pooled SDR **(C)** and PDR **(D)** were reversed between the two groups.

We synthesized CR, PR, ORR, SDR, DCR, and PDR to assess the efficacy of copanlisib monotherapy or its combination with rituximab for patients with all R/R B-NHL. All enrolled studies reported CR, PR, and ORR for patients treated with copanlisib-containing regimens. The pooled CR, PR and ORR were 13% (95% CI: 4%-23%), 40% (95% CI: 32%-50%), and 57% (95% CI: 46%-71%), respectively. For copanlisib monotherapy, the pooled CR and ORR were 6% (95% CI: 1%-12%) and 42% (95% CI: 30%-59%), respectively. For combination therapy, including copanlisib plus rituximab, the pooled CR and ORR were 34% (95% CI: 29%-39%) and 89% (95% CI: 77%-100%), respectively. The above subgroup analysis suggested that the CR and ORR of combination therapy, including copanlisib plus rituximab, were higher than those of copanlisib monotherapy for R/R B-NHL (34% vs. 6%, p<0.01; 89% vs. 42%, p<0.01) (**Figure 2**). There was no significant difference in PR between copanlisib monotherapy and combination therapy, including copanlisib plus rituximab (**Supplementary Figure S1**). The SDR, DCR and PDR were shown in seven articles, which were 19% (95% CI: 10%-29%), 86% (95% CI: 78%-94%), and 9% (95% CI: 3%-14%), respectively. However, the copanlisib monotherapy subgroup displayed a higher SDR and PDR than the combination therapy subgroup, including copanlisib plus rituximab (25% vs. 9%, p<0.01; 16% vs. 2%, p=0.02) (**Figure 2**). No significant difference occurred for DCR between copanlisib monotherapy and combination therapy, including copanlisib plus rituximab (**Supplementary Figure S1**).

**Figure 3:**
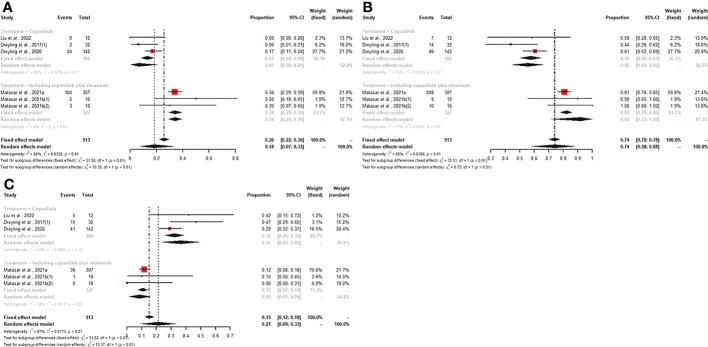
The pooled CR **(A)** and ORR **(B)** in patients with R/R indolent B-NHL receiving copanlisib monotherapy were significantly lower than those receiving combination therapy, including copanlisib plus rituximab, while result for pooled SDR **(C)** was reversed between the two groups.

For patients with R/R indolent B-NHL, six studies reported all efficacy data (CR, PR, ORR, SDR, DCR, and PDR) with copanlisib monotherapy or copanlisib plus rituximab. The pooled CR, PR, ORR, SDR, DCR and PDR were 18% (95% CI: 7%-33%), 44% (95% CI: 40%-49%), 74% (95% CI: 58%-88%), 21% (95% CI: 9%-33%), 91% (95% CI: 88%-93%), and 2% (95% CI: 1%-3%), respectively. Subgroup analysis of CR and ORR showed that both CR and ORR were lower in patients with R/R indolent B-NHL receiving copanlisib monotherapy than in those receiving combination therapy, including copanlisib plus rituximab (7% vs. 34%, p<0.01; 58% vs. 92%, p<0.01) (**Figure 3**). The SDR in the copanlisib monotherapy subgroup was higher than that in the combination therapy subgroup, including copanlisib plus rituximab (32% vs. 11%, p<0.01) (**Figure 3**). The difference in PR, DCR, and PDR between copanlisib monotherapy and combination therapy, including copanlisib plus rituximab, was not statistically significant (**Supplementary Figure S2**).

**Figure 4:**
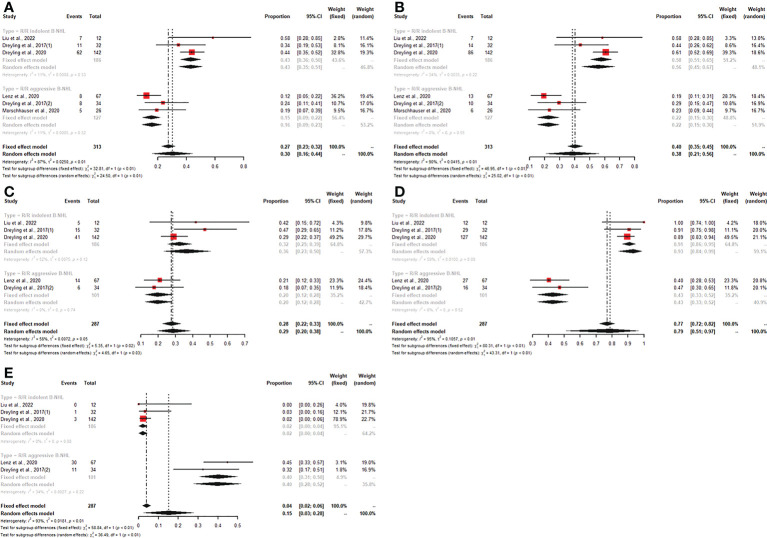
The pooled PR **(A)**, ORR **(B)**, SDR **(C)**, and DCR **(D)** in patients with R/R indolent B-NHL receiving copanlisib monotherapy were significantly higher than those in patients with R/R aggressive B-NHL receiving copanlisib monotherapy, while result for pooled PDR **(E)** was reversed between the two groups.

For patients receiving copanlisib monotherapy, six trials included patients with R/R indolent B-NHL or R/R aggressive B-NHL, and all reported CR, PR, and ORR. The pooled CR, PR, and ORR were 6% (95% CI: 0-12%), 30% (95% CI: 16%-44%), and 38% (95% CI: 21%-56%), respectively. The subgroup analysis showed that patients with R/R indolent B-NHL treated with copanlisib monotherapy had higher PR and ORR than patients with R/R aggressive B-NHL (43% vs. 15%, p<0.01; 58% vs. 22%, p<0.01) (**Figure 4**). No significant difference existed in CR between the R/R indolent B-NHL subgroup and the R/R aggressive B-NHL subgroup (**Supplementary Figure S3**). Five studies displayed SDR, DCR, and PDR. The pooled SDR, DCR and PDR were 29% (95% CI: 20%-38%), 79% (95% CI: 51%-97%), and 15% (95% CI: 3%-28%), respectively. In the R/R indolent B-NHL subgroup, the SDR and DCR were higher than those in the R/R aggressive B-NHL subgroup (36% vs. 20%, p=0.03; 93% vs. 43%, p<0.01) (**Figure 4**), while the PDR was lower than that in the R/R aggressive B-NHL subgroup (2% vs. 40%, p<0.01) (**Figure 4**).

Of all included studies, only four described the survival outcomes, of which three reported median PFS and OS with copanlisib monotherapy and one showed median PFS with combination therapy, including copanlisib plus rituximab. The best survival outcomes with copanlisib monotherapy for patients with R/R B-NHL were a median PFS of 12.5 months and a median OS of 42.6 months (**Table 1**). Due to incomplete data, the survival outcomes were not further synthesized.

### Safety

**Figure 5:**
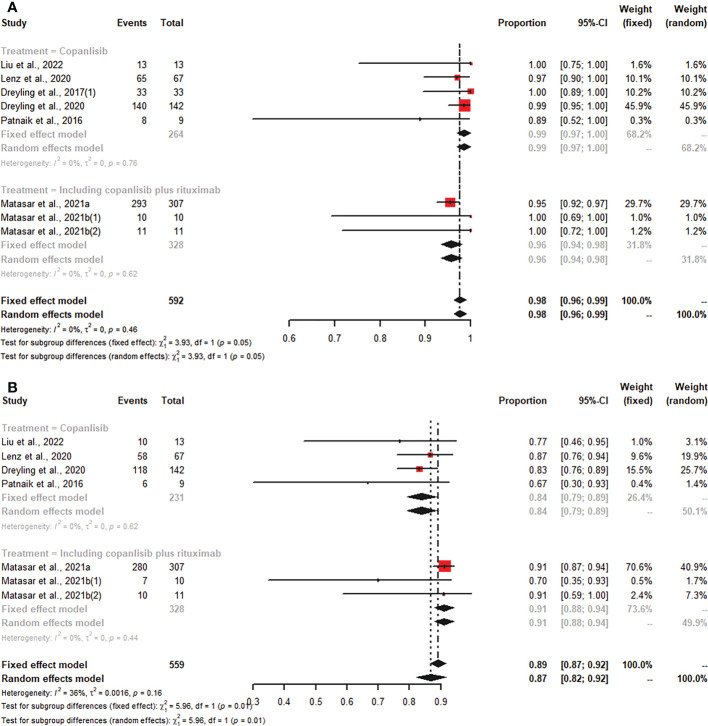
The pooled risk of any grade **(A)** and grade ≥3 **(B)** AEs in patients with R/R B-NHL receiving copanlisib monotherapy or combination therapy with rituximab.

Of all studies, seven studies reported any grade AEs, and six articles described grade ≥3 AEs. In patients with R/R B-NHL who were treated with copanlisib monotherapy, the pooled risks of any grade and grade ≥3 AEs were 99% (95% CI: 97%-100%) and 84% (95% CI: 79%-89%), respectively (**Figure 5**). Patients with R/R B-NHL receiving combination therapy, including copanlisib plus rituximab, had a 96% (95% CI: 94%-98%) risk of any grade AEs and a 91% (95% CI: 88%-94%) risk of grade ≥3 AEs (**Figure 5**). For all patients with R/R B-NHL, the difference in any grade AEs was not statistically significant between copanlisib monotherapy and combination therapy, including copanlisib plus rituximab (99% vs. 96%, p=0.05; **Figure 5**). However, the pooled risk of grade ≥3 AEs for the combination therapy, including copanlisib plus rituximab, was significantly higher than that for copanlisib monotherapy (91% vs. 84%, p=0.01; **Figure 5**).

For all R/R B-NHL patients treated with copanlisib monotherapy or in combination with rituximab, the common any grade AEs included hyperglycemia (66.75%), hypertension (48.57%), diarrhea (35.06%), nausea (34.98%) and fatigue (30.33%) (**Table 2**). The common grade ≥3 AEs included hyperglycemia (45.14%), hypertension (35.07%), neutropenia (14.75%), pneumonia (7.03%), and diarrhea (5.09%) (**Table 2**). For all R/R B-NHL patients treated with copanlisib monotherapy, the common any grade toxicities were hyperglycemia (63.69%), hypertension (49.69%), diarrhea (35.94%), nausea (35.8%) and fatigue (32.76%). Hyperglycemia (37.61%), hypertension (27.57%), and neutropenia (18.01%) were the common grade ≥3 AEs in copanlisib monotherapy. For all R/R B-NHL patients treated with copanlisib plus rituximab, the common any grade toxicities included hyperglycemia (69.93%), hypertension (47.89%), nausea (40.53%), and decreased platelet count (38.87%), and the common grade ≥3 AEs included hyperglycemia (56.41%), hypertension (39.34%), and neutropenia (9.36%). Other AEs in patients with R/R B-NHL receiving copanlisib monotherapy or combination therapy with rituximab are listed in **Supplementary Table S2**.

**Table 2:** The incidence of adverse events in any grade or grade ≥3 for patients with R/R B-NHL.

AEs	Treatment	Any grade					Grade ≥3				
		Included study	Event	Total patients	Pooled rate(95% Cl)	*p-value*	Included study	Event	Total patients	Pooled rate(95% Cl)	*p-value*
Hematological												
Neutropenia	Copanlisib	3	59	242	0.2278[0.1297;0.3440]	P=0.2661	2	42	209	0.1801[0.0782;0.3124]	P=0.1811
	Including copanlisib plus rituximab	2	66	328	0.1067[0.0043;0.3190]		2	50	328	0.0936[0.0067;0.2645]	
	Overall	5	125	570	0.1899[0.1202;0.2711]		4	92	537	0.1475[0.0843;0.2246]	
Decreased platelet count	Copanlisib	3	27	188	0.1436[0.0973;0.1972]	P=0.7445	2	7	155	0.0435[0.0100;0.0769]	P=0.5806
	Including copanlisib plus rituximab	2	52	328	0.3887[0.0770;0.7655]		2	10	328	0.0440[0.0000;0.1109]	
	Overall	5	79	516	0.2101[0.1249;0.3105]		4	17	483	0.0281[0.0132;0.0429]	
Non-hematological												
Fatigue	Copanlisib	4	75	251	0.3276[0.2354;0.4557]	P<0.0001	2	4	209	0.0190[0.0050;0.0419]	P=0.2762
	Including copanlisib plus rituximab	2	52	328	0.2828[0.1143;0.6994]		2	5	328	0.0143[0.0043;0.0299]	
	Overall	6	127	579	0.3033[0.2038;0.4513]		4	9	537	0.0160[0.0072;0.0284]	
Diarrhea	Copanlisib	5	95	264	0.3594[0.3015;0.4172]	P=0.7627	3	13	222	0.0289[0.0001;0.1058]	P=0.8811
	Including copanlisib plus rituximab	2	113	328	0.3437[0.2925;0.3949]		2	17	328	0.0514[0.0302;0.0779]	
	Overall	7	208	592	0.3506[0.3122;0.3889]		5	30	550	0.0509[0.0341;0.0708]	
Nausea	Copanlisib	4	71	251	0.3580[0.2117;0.5042]	P=0.3357	2	2	209	0.0086[0.0000;0.0284]	P=0.6445
	Including copanlisib plus rituximab	2	80	328	0.4053[0.1214;0.6892]		2	2	328	0.0000[0.0000;0.0019]	
	Overall	6	151	579	0.3498[0.2505;0.4491]		4	4	537	0.0000[0.0000;0.0053]	
Pneumonia	Copanlisib	2	25	175	0.1403[0.0912;0.1972]	P=0.9685	1	12	142	0.1056	_
	Including copanlisib plus rituximab	2	45	328	0.1175[0.0806;0.1590]		2	21	328	0.0610[0.0352;0.0869]	
	Overall	4	70	503	0.1261[0.0959;0.1591]		3	33	470	0.0703[0.0472;0.0933]	
Hyperglycemia	Copanlisib	5	136	264	0.6369[0.4379;0.9264]	P<0.0001	4	87	231	0.3761[0.3148;0.4394]	P<0.0001
	Including copanlisib plus rituximab	2	228	328	0.6993[0.6513;0.7508]		2	185	328	0.5641[0.5101;0.6173]	
	Overall	7	364	592	0.6675[0.5399;0.8254]		6	272	559	0.4514[0.5101;0.6173]	
Hypertension	Copanlisib	5	106	264	0.4969[0.3045;0.6898]	P=0.0368	4	66	231	0.2757[0.2168;0.3384]	P=0.0074
	Including copanlisib plus rituximab	2	161	328	0.4789[0.2664;0.6950]		2	131	328	0.3934[0.3380;0.4502]	
	Overall	7	267	592	0.4857[0.3604;0.6118]		6	197	559	0.3507[0.2576;0.4492]	

For all R/R B-NHL patients, the comparison of AEs between the copanlisib monotherapy group and the combination therapy, including copanlisib plus rituximab group, suggested that significant differences existed in any grade of hyperglycemia (63.69% vs. 69.93%, p<0.0001), hypertension (49.69% vs. 47.89%, p=0.0368) and fatigue (32.76% vs. 28.28%, p<0.0001) between the two groups, and significant differences were shown in grade ≥3 hypertension (37.61% vs. 56.41%, p<0.0001) and hyperglycemia (27.57% vs. 39.34%, p=0.0074) between the two groups. The above results are shown in **Table 2**.

### Study quality

**Figure 6:**
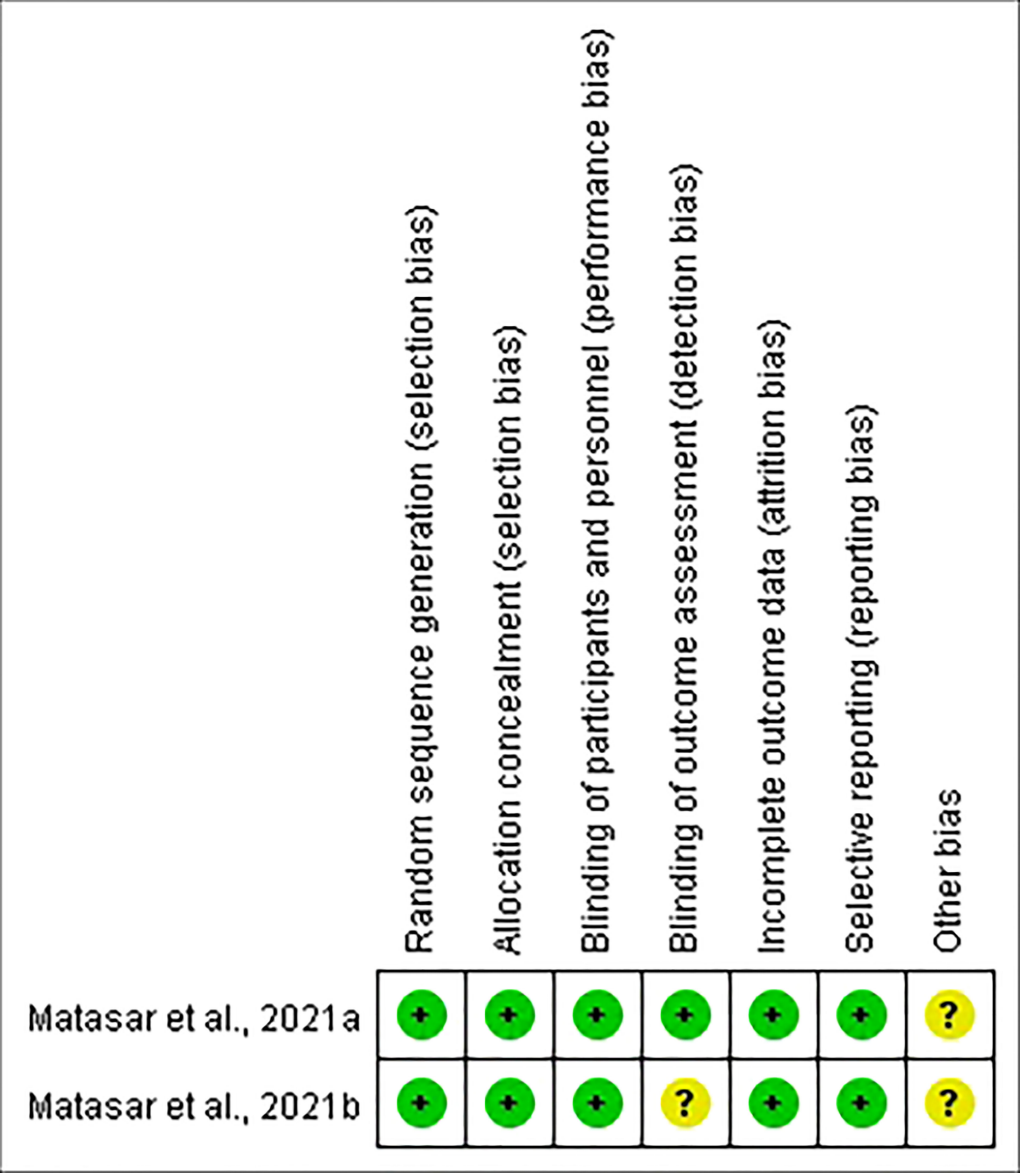
Quality assessment of the randomized studies.

Six of the studies were open-label, and two were double-blind. MINORS was applied to evaluate the quality of the six nonrandomized trials. The scores for each article ranged from 11 to 13 (**Table 3**). Meanwhile, the quality of the two RCTs was estimated by the Cochrane Collaboration’s Risk of Bias Tool. The quality assessment of the included RCTs was good (**Figure 6**). Therefore, the quality of the enrolled studies was not poor.

**Table 3:** Quality assessment of the non-randomized studies.

Reference	Study aims	Consecutive patient inclusion criteria	Prospective collection of data	Endpoint consistent with the study aim	Unbiased evaluation of endpoints	Follow-up period	Loss to follow-up less than 5%	Prospective calculation of the sample size	Total
Liu et al., 2022 (27)	2	2	2	2	0	2	2	0	12
Lenz et al., 2020 (28)	2	2	2	2	0	2	2	0	12
Dreyling et al., 2017 (29)	2	2	2	2	1	2	2	0	13
Dreyling et al., 2020 (23)	2	2	2	2	1	2	2	0	13
Patnaik et al., 2016 (30)	2	2	2	1	0	2	2	0	11
Morschhauser et al., 2020 (31)	2	2	2	1	0	2	2	0	11

0. not reported.

1. reported but inadequate.

2. reported and adequate.

## Discussion

R/R B-NHL indicates a poor prognosis. Patients with R/R B-NHL have often received multiple lines of treatment and have poor responses to various treatment regimens. Therefore, there is an urgent need to find effective treatments for patients with R/R lymphoma. Copanlisib is active against all four isoforms; however, it has higher targeting of PI3Kα and PI3Kδ. We conducted a meta-analysis to evaluate the efficacy and safety of copanlisib monotherapy or combination therapy, including copanlisib plus rituximab, for patients with R/R B-NHL.

Our analysis showed that among patients receiving monotherapy, the pooled ORRs of patients with R/R B-NHL, R/R indolent B-NHL and R/R aggressive B-NHL were 42%, 58%, and 22%, respectively. The results indicated that copanlisib had promising efficacy in patients with R/R B-NHL who failed to respond to previous antitumor therapy. The number of lines of previous anticancer therapy the participant had received was from 1 to 13, mainly including rituximab, alkylating agents, high-dose chemotherapy/autologous stem cell transplant, radioimmunotherapy, and so on. The efficacy results of the patients with R/R B-NHL receiving copanlisib monotherapy were similar to the efficacy results of a meta-analysis that enrolled five clinical trials involving a total of 331 NHL patients receiving copanlisib, 174 of whom were indolent and 115 of whom were aggressive (33). In addition, similar ORRs have been reported for other PI3K inhibitors (duvelisib and idelalisib) approved for the treatment of lymphoma. Previous studies showed that the ORRs of patients with lymphoma receiving duvelisib were 47.3%-58.1% (34–36), and the ORRs of patients with lymphoma receiving idelalisib were 40%-57% (37–39).

PI3K is essential in the BCR signaling pathway. Dysregulation of PI3K is directly associated with the development of cancer, while abnormal activation of class I PI3K is related to acquired drug resistance (40). PI3Kδ is only expressed in hematopoietic cells and is usually expressed in B-cell malignancies (41). Copanlisib is highly selective for PI3Kα and PI3Kδ, so it can play a good role in treating B-NHL. In addition, the subgroup analysis revealed that copanlisib monotherapy had better efficacy in patients with R/R indolent B-NHL than in those with R/R aggressive B-NHL. Aggressive NHL is difficult to control once relapsed or refractory (10).

The results of subgroup analysis showed that combination therapy, including copanlisib plus rituximab, had a higher effect (CR and ORR) than copanlisib monotherapy for R/R B-NHL and R/R indolent B-NHL. At the same time, patients with R/R B-NHL receiving combination therapy had lower PDR. The above results suggest that combination therapy of copanlisib plus rituximab is a promising regimen for patients with R/R B-NHL. Rituximab, a CD20 monoclonal antibody, is often the first-choice treatment for patients with B-NHL and is one of the standard options for patients with R/R B-NHL (42). PI3K plays a part in the development of B-cell lymphoma. A few previous studies have investigated the efficacy of other PI3K inhibitors in combination with rituximab for NHL. The results of a phase I study showed that the ORR of idelalisib plus rituximab in patients with R/R indolent NHL was 75% (43). Flinn IW et al. revealed that duvelisib plus rituximab had an ORR of 71.4% in patients with NHL (44). The ORR of the above studies seemed to be lower than that of our meta-analysis (89%). However, there are few relevant trials of copanlisib/idelalisib/duvelisib in combination with rituximab for patients with lymphoma. Meanwhile, there were only 2 studies on combination therapy included in our analysis, so more prospective clinical trials about combination therapy, including copanlisib plus rituximab, for lymphoma are needed.

Our meta-analysis suggested that the common any grade AEs included hyperglycemia, hypertension, diarrhea, nausea, and fatigue. The common grade ≥3 AEs included hyperglycemia, hypertension, neutropenia, pneumonia, and diarrhea. It is worth noting that the most common AEs, whether of any grade or grade ≥3, were hyperglycemia and hypertension. Hyperglycemia was also found to be a common AE in clinical trials of other PI3K inhibitors (45, 46). Alterations in PI3K signaling play a role in the development of noninsulin-dependent diabetes (47). Hyperglycemia induced by copanlisib may be related to the targeting of PI3Kα inhibition (23). Hyperglycemia often occurs during intravenous infusion of copanlisib and is often transient and controllable. In most patients, blood sugar levels can be normalized with fluid replacement (48). Before using copanlisib, it is recommended to screen the patient for diabetes, and if the patient is diagnosed with diabetes, they can receive copanlisib until their blood glucose is adequately controlled (49). If the blood sugar level of the patient is not effectively controlled, it is best to switch to other drugs that do not affect blood sugar.

In studies of other PI3K inhibitors, hypertension has been less frequently reported as an AE (48). The mechanism by which copanlisib causes hypertension is unclear, but it may be related to the interaction of PI3Kγ and angiotensin II (47). Hypertension often occurs during intravenous infusion and is usually transient and manageable. During an infusion of copanlisib, the patient’s blood pressure should be closely monitored. If the patient’s blood pressure continues to rise, antihypertensive drugs can be given appropriately, and the dose of copanlisib should be reduced or discontinued if necessary (50).

Diarrhea is also a common AE that is usually less than grade 3 and can be relieved by dietary or drug therapy (47). Nausea and fatigue were also mostly mild and could be alleviated with medication or rest. Compared with idelalisib and duvelisib, copanlisib exhibited less gastrointestinal toxicity, possibly related to its intermittent intravenous infusion (51). Hematological toxicities caused by copanlisib, including neutropenia, decreased platelet count, anemia, etc., may be related to the suppression of the bone marrow by copanlisib (50). Patients should have their blood monitored during the use of copanlisib, and severe hematological toxicities can be managed by reducing or discontinuing copanlisib. Pneumonia is a common infection induced by copanlisib. Patients using copanlisib should be closely monitored for symptoms and signs related to infection. For infections of grade 3 or higher, it is recommended to discontinue copanlisib treatment and actively take anti-infective treatment (50).

The risk of other AEs of special interest, including increased ALT/AST and rash, in patients with lymphoma using copanlisib was lower than that in patients with lymphoma using idelalisib or duvelisib (34, 35, 37, 39). This suggested that copanlisib may have superior safety for the liver and skin. With copanlisib, ALT and AST levels should be monitored closely. If ALT/AST exceeds 5 times the upper limit, copanlisib should be stopped temporarily, and a reduced dose of copanlisib should be restarted after the ALT/AST returns to normal. When severe liver toxicity occurs, copanlisib should be permanently discontinued (52). Patients with severe or grade ≥3 cutaneous reactions during the use of copanlisib should consult a dermatologist to evaluate their need for medication (47). The comparison of the incidence of AEs between copanlisib and other PI3K inhibitors (idelalisib and duvelisib) was shown in **Supplementary Table S3**.

Previous studies have revealed that patients with lymphoma receiving duvelisib have a 99%-100% risk of any grade AEs and an 88.4%-87% risk of grade ≥3 AEs (34, 53, 54). This was similar to our analysis of the risk of any grade (99%) and grade ≥3 (84%) AEs in patients with R/R B-NHL receiving copanlisib monotherapy. However, compared with our results, patients with lymphoma using idelalisib had a lower risk of any grade (82%-98.6%) and grade ≥3 (54%-65.3%) AEs (37, 38, 55). PI3kα and PI3Kβ are expressed in various kinds of cells, while PI3Kγ and PI3Kδ are mainly expressed in the hemopoietic system (16). The incidence of AEs with idelalisib was lower than that with copanlisib and duvelisib, which may be because idelalisib is an isoform-specific inhibitor and only has targeting activity for PI3Kδ.

In recent years, serious safety concerns about idelalisib and duvelisib have attracted significant attention. The FDA gave a black box warning for the AEs caused by these two drugs (56, 57). Idelalisib mainly leads to serious or fatal hepatotoxicity, diarrhea/colitis, pneumonitis, infections, and intestinal perforation, and duvelisib mostly causes serious or fatal diarrhea/colitis, cutaneous reactions, infections, and pneumonitis (49). Compared to the serious toxicities caused by idelalisib and duvelisib (21), copanlisib seems to have manageable safety.

This meta-analysis revealed that the risk of grade ≥3 AEs in combination therapy, including copanlisib plus rituximab, was higher than that in copanlisib monotherapy. The difference in grade ≥3 AEs between the two groups was mainly reflected in hyperglycemia and hypertension. However, these AEs, which were significantly different between the two groups, were manageable. Therefore, the AEs in patients with R/R B-NHL receiving either copanlisib monotherapy or combination therapy, including copanlisib plus rituximab, were tolerable.

Our study has several limitations. First, the number of articles included in our analysis was limited. Second, most of the involved clinical trials were single-arm trials. Third, the dose of copanlisib varied among the studies. All of the above may cause bias. Similarly, due to the inconsistent follow-up times and incomplete data among the articles, our meta-analysis did not conduct a synthetic analysis of the survival outcomes.

In conclusion, our meta-analysis demonstrated that the efficacy of both copanlisib monotherapy and combination therapy, including copanlisib plus rituximab, in patients with R/R B-NHL was satisfactory, while treatment-related AEs were tolerable. Compared with copanlisib monotherapy, combination therapy of copanlisib plus rituximab showed superior efficacy for treating R/R B-NHL, and its safety was manageable. Furthermore, this research revealed that copanlisib monotherapy had better efficacy for patients with R/R indolent B-NHL than for patients with R/R aggressive B-NHL. The efficacy and safety of copanlisib needs to be compared with other drugs for treating lymphoma and there is a need to explore the efficacy and safety of copanlisib-based combination therapy for patients with lymphoma further.

## Data availability statement

The original contributions presented in the study are included in the article/**Supplementary Material**. Further inquiries can be directed to the corresponding author.

## Author contributions

JW collected, analyzed the data and wrote the article. JW, HZ and MM performed the statistical analysis. AZ and ZC analyzed and interpreted the data. LL and MW prepared the pictures and tables. TN and HZ provided the idea and modified the article. All authors read and approved the final manuscript. All authors contributed to the article and approved the submitted version.

## Funding

This work was supported by Incubation Program for Clinical Trials (No. 19HXFH030), and 1.3.5 Project for Disciplines of Excellence, West China Hospital, Sichuan University (No. ZYJC21007).

## Conflict of interest

The authors declare that the research was conducted in the absence of any commercial or financial relationships that could be construed as a potential conflict of interest.

## Publisher’s note

All claims expressed in this article are solely those of the authors and do not necessarily represent those of their affiliated organizations, or those of the publisher, the editors and the reviewers. Any product that may be evaluated in this article, or claim that may be made by its manufacturer, is not guaranteed or endorsed by the publisher.
