# Supplementary material for: Efficacy and safety of copanlisib in relapsed/refractory B-cell non-Hodgkin lymphoma: A meta-analysis of prospective clinical trials
Source: Front Immunol. 2022 Nov 11;13:1034253. doi: 10.3389/fimmu.2022.1034253 (PMC9691663; doi:10.3389/fimmu.2022.1034253)
Supplement: Supplementary file 1 [file DataSheet_1.docx]

**Supplementary material**

**Methods**

***Definitions of efficacy variables***

Rate of stable disease (SDR) was defined as the proportion of patients with stable disease who did not achieve a complete response or partial response. Overall response rate (ORR) was defined as the proportion of patients who have a complete response, or partial response. Disease control rate (DCR) was defined as the proportion of patients who had a complete response, partial response, or stable disease. Rate of progressive disease (PDR) was defined as the proportion of patients from the start of treatment to disease progression or death associated with disease progression. Progression-free survival (PFS) was defined as the time from first medication (copanlisib) until disease progression or death. Overall survival (OS) was defined as the time from first medication (copanlisib) until death.

**Table S1.** Other characteristics of included studies.

| **N0.** | **Study** | **Dose of copanlisib** | **Way of administration** | **Median duration of treatment** | **Follow-up time** | **Reduction or interruption or delay of doses due to adverse events (n)** | **Discontinued treatment due to adverse events (n)** |
| --- | --- | --- | --- | --- | --- | --- | --- |
| 1 | Liu et al., 2022(1) | 60mg, days 1, 8, and 15 of a 28-day cycle | Intravenous infusion | 15 weeks | — | 4 | 2 |
| 2 | Lenz et al., 2020(2) | 60mg, days 1, 8, and 15 of a 28-day cycle | Intravenous infusion | — | — | 43 | 17 |
| 3 | Dreyling et al., 2017(1)(3) | 0.8mg / kg, days 1, 8, and 15 of a 28-day cycle | Intravenous infusion | 22.7 weeks | — | 61 | 21 |
|  | Dreyling et al., 2017(2)(3) |  |  | 8 weeks |  |  |  |
| 4 | Dreyling et al., 2020(4) | 60mg, day 1, 8, and 15 of a 28-day cycle | Intravenous infusion | 26 weeks | 2 years | 137 | 38 |
| 5 | Patnaik et al., 2016(5) | 0.8mg / kg, days 1, 8, and 15 of a 28-day cycle | Intravenous infusion | 6 weeks | — | — | — |
| 6 | Morschhauser et al., 2020(6) | 0.4 or 0.8mg / kg, days 1, 8, and 15 of a 28-day cycle | Intravenous infusion | 7 weeks | — | — | — |
| 7 | Matasar et al., 2021a(7) | 60mg, days 1, 8, and 15 of a 28-day cycle | Intravenous infusion | 33.2 weeks | 19.2 months | — | 104 |
| 8 | Matasar et al., 2021b (1)(8) | 45 or 60mg, days 1, 8, and 15 of a 28-day cycle | Intravenous infusion | 12 weeks | — | — | 4 |
|  | Matasar et al., 2021b (2)(8) |  |  | 32 weeks | — | — | 5 |


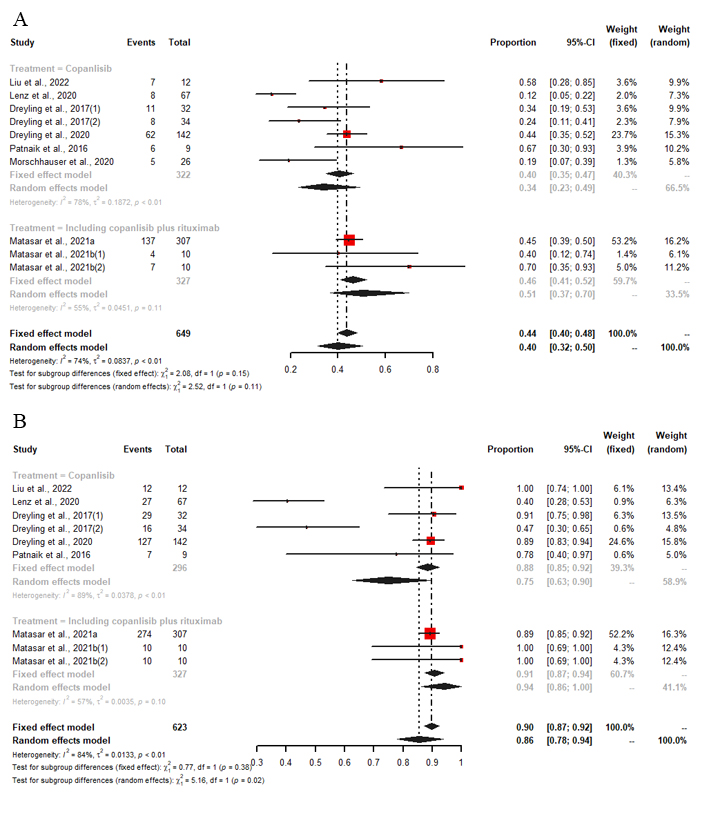


**Figure S1.** There was no significant difference in the pooled PR (A) and DCR (B) between patients with R/R B-NHL receiving copanlisib monotherapy and those receiving combination therapy, including copanlisib plus rituximab.


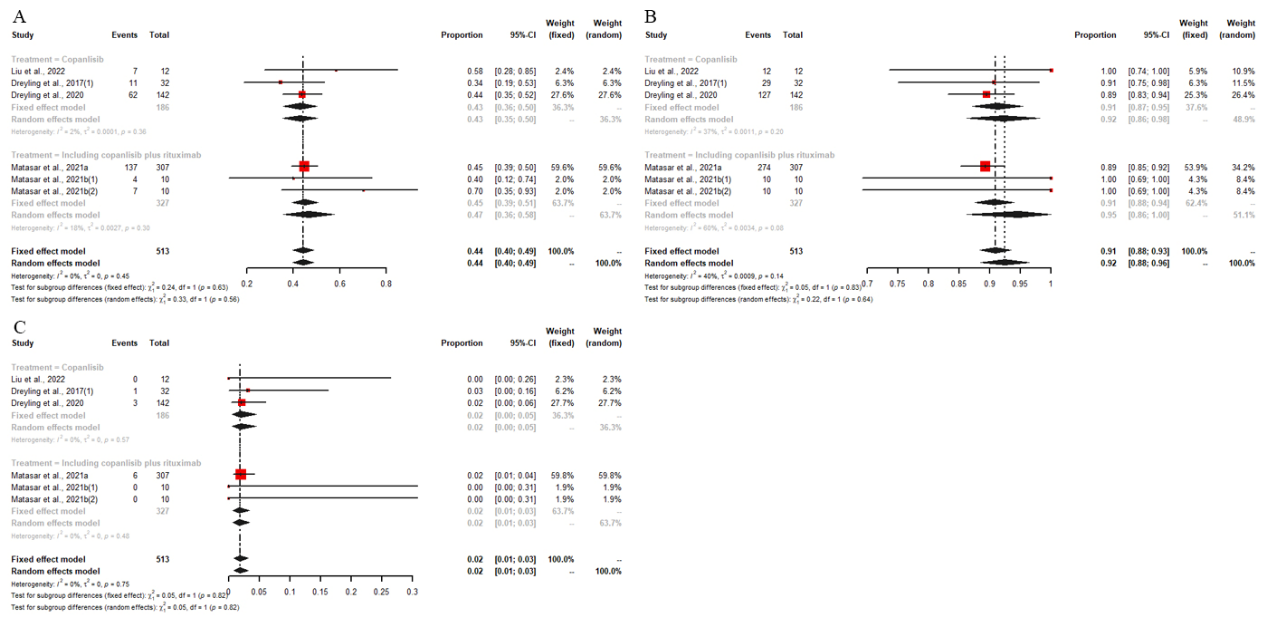


**Figure S2.** There was no significant difference in the pooled PR (A), DCR (B), and PDR (C) between patients with R/R indolent B-NHL receiving copanlisib monotherapy and those receiving combination therapy, including copanlisib plus rituximab.


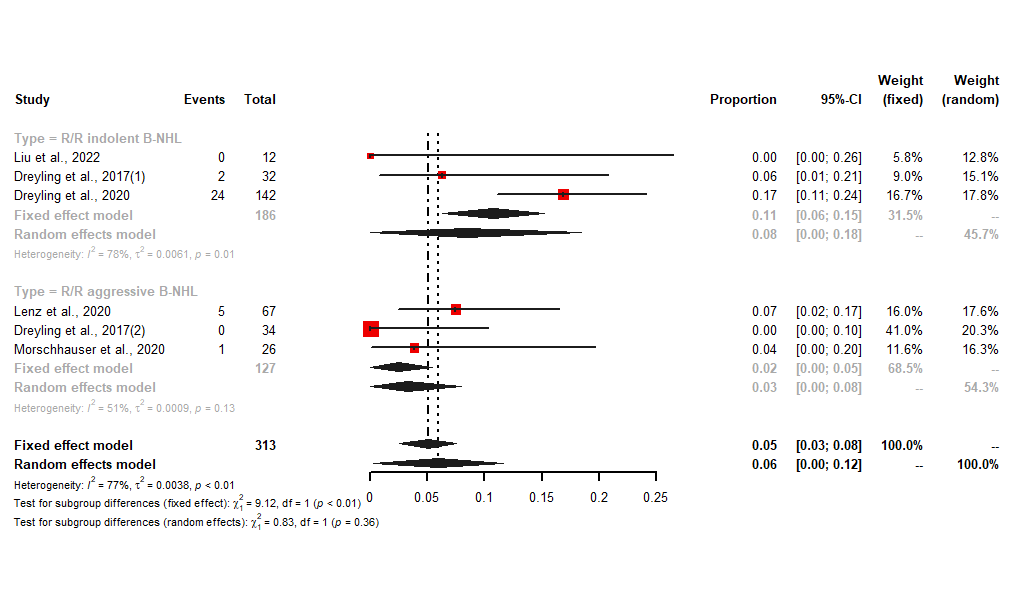


**Figure S3.** There was no significant difference in the pooled CR between patients with R/R indolent B-NHL receiving copanlisib monotherapy and those with R/R aggressive B-NHL receiving copanlisib monotherapy.

**Table S2.** **The incidence of other adverse events in any grade or grade ≥3.**

| AEs | Treatment | Any grade | | | | |  | Grade ≥3 | | | | |
| --- | --- | --- | --- | --- | --- | --- | --- | --- | --- | --- | --- | --- |
|  |  | Included study | Event | Total patients | Pooled rate (95% Cl) | *p-value* |  | Included study | Event | Total patients | Pooled rate (95% Cl) | *p-value* |
| Hematological | | | | | | | | | | | | |
| Anemia | Copanlisib | 3 | 38 | 184 | 0.2188[0.0950;0.3713] | P=1 |  | 2 | 8 | 151 | 0.0523[0.0226;0.0934] | P=0.9086 |
|  | Including copanlisib plus rituximab | 2 | 67 | 328 | 0.2965[0.1115;0.5191 |  |  | 2 | 15 | 328 | 0.0442[0.0246;0.0690] |  |
|  | Overall | 5 | 105 | 512 | 0.2284[0.1524;0.3132] |  |  | 4 | 23 | 479 | 0.0467[0.0296;0.0673] |  |
| Non-hematological | | | | | | | | | | | | |
| Pyrexia | Copanlisib | 4 | 69 | 255 | 0.2649[0.2107;0.3227] | P=0.1831 |  | 3 | 7 | 222 | 0.0255[0.0042;0.0468] | P=0.2395 |
|  | Including copanlisib plus rituximab | 2 | 72 | 328 | 0.2991[0.1267;0.5026] |  |  | 2 | 5 | 328 | 0.0158[0.0019;0.0298] |  |
|  | Overall | 6 | 141 | 583 | 0.2307[0.1954;0.2677] |  |  | 5 | 12 | 550 | 0.0187[0.0071;0.0304] |  |
| Headache | Copanlisib | 2 | 16 | 100 | 0.1658[0.1061;0.2591] | P=0.861 |  | 1 | 0 | 67 | 0 | — |
|  | Including copanlisib plus rituximab | 2 | 48 | 328 | 0.1525[0.1177;0.1977] |  |  | 2 | 2 | 328 | 0.0064[0.0000;0.0154] |  |
|  | Overall | 4 | 64 | 428 | 0.1558[0.1245;0.1950] |  |  | 3 | 2 | 395 | 0.0054[0.0000;0.0136] |  |
| Decreased appetite | Copanlisib | 3 | 30 | 242 | 0.1232[0.0849;0.1674] | — |  | 2 | 0 | 209 | 0 | — |
|  | Including copanlisib plus rituximab | 1 | 2 | 21 | 0.0517[0.0000;0.3990] |  |  | 1 | 0 | 21 | 0 |  |
|  | Overall | 5 | 32 | 263 | 0.1160[0.0802;0.1574] |  |  | 3 | 0 | 230 | 0.0000[0;0.0003] |  |
| Vomiting | Copanlisib | 4 | 38 | 251 | 0.1510[0.1095;0.1978] | P=0.8772 |  | 2 | 1 | 209 | 0.0015[0.0000;0.0107] | P=1 |
|  | Including copanlisib plus rituximab | 2 | 47 | 328 | 0.0897[0.0047;0.2641] |  |  | 2 | 1 | 328 | 0.0032[0.0000;0.0096] |  |
|  | Overall | 6 | 85 | 579 | 0.1441[0.1167;0.1738] |  |  | 4 | 2 | 537 | 0.0027[0.0000;0.0079] |  |
| Constipation | Copanlisib | 3 | 34 | 242 | 0.1382[0.0963;0.1858] | — |  | 2 | 0 | 209 | 0.0000[0;0.0085] | — |
|  | Including copanlisib plus rituximab | 1 | 6 | 21 | 0.2856 |  |  | 1 | 0 | 21 | 0 |  |
|  | Overall | 4 | 40 | 263 | 0.1398[0.0971;0.1878] |  |  | 3 | 0 | 230 | 0.0000[0;0.0003] |  |
| Cough | Copanlisib | 4 | 47 | 255 | 0.1840[0.1364;0.2315] | P=0.3612 |  | 3 | 1 | 222 | 0.0015[0.0000;0.0106] | P=0.4036 |
|  | Including copanlisib plus rituximab | 2 | 50 | 328 | 0.1480[0.0000;0.3059] |  |  | 2 | 0 | 328 | 0.0000[0.0000;0.0045] |  |
|  | Overall | 6 | 97 | 583 | 0.1556[0.1262;0.1849] |  |  | 5 | 1 | 550 | 0.0003[0.0000;0.0043] |  |
| Upper respiratory tract infection | Copanlisib | 2 | 23 | 175 | 0.1123[0.0410;0.2127] | P= 0.2025 |  | 1 | 2 | 142 | 0.0141 | _ |
|  | Including copanlisib plus rituximab | 2 | 59 | 328 | 0.1183[0.0085;0.3281] |  |  | 2 | 3 | 328 | 0.0086[0.0015;0.0214] |  |
|  | Overall | 4 | 82 | 503 | 0.1263[0.0657;0.2032] |  |  | 3 | 5 | 470 | 0.0101[0.0031;0.0211] |  |
| Dyspnea | Copanlisib | 2 | 11 | 100 | 0.1099[0.0564;0.1783] | P=0.1512 |  | 1 | 3 | 67 | 0.0448 | _ |
|  | Including copanlisib plus rituximab | 2 | 20 | 328 | 0.0582[0.0355;0.0861] |  |  | 2 | 2 | 328 | 0.0000[0.0000;0.0019] |  |
|  | Overall | 4 | 31 | 428 | 0.0689[0.0469;0.0948] |  |  | 3 | 5 | 395 | 0.0001[0.0000;0.0084] |  |
| ALT increased | Copanlisib | 2 | 9 | 155 | 0.1011[0.0000;0.2722] | P=0.3843 |  | 2 | 1 | 155 | 0.0059[0.0000;0.0240] | P=1 |
|  | Including copanlisib plus rituximab | 2 | 28 | 328 | 0.0747[0.0000;0.1660] |  |  | 2 | 4 | 328 | 0.0114[0.0028;0.0258] |  |
|  | Overall | 4 | 37 | 483 | 0.0667[0.0205;0.1128] |  |  | 4 | 5 | 328 | 0.0095[0.0028;0.0200] |  |
| AST increased | Copanlisib | 1 | 3 | 142 | 0.0211 | — |  | 1 | 0 | 142 | 0 | — |
|  | Including copanlisib plus rituximab | 2 | 28 | 328 | 0.0747[0.0000;0.1660] |  |  | 2 | 4 | 328 | 0.0127[0.0002;0.0253] |  |
|  | Overall | 3 | 31 | 470 | 0.0514[0.0000;0.1067] |  |  | 3 | 4 | 470 | 0.0048[0.0000;0.0124] |  |
| Rash | Copanlisib | 3 | 17 | 109 | 0.1521[0.0849;0.2193] | P=0.4537 |  | 2 | 2 | 76 | 0.0168[0.0000;0.0456] | P=0.09214 |
|  | Including copanlisib plus rituximab | 2 | 40 | 328 | 0.1168[0.0822;0.1513] |  |  | 2 | 1 | 328 | 0.0032[0.0000;0.0096] |  |
|  | Overall | 5 | 57 | 437 | 0.1242[0.0934;0.1549] |  |  | 4 | 3 | 404 | 0.0039[0.0000;0.0101] |  |
| Oral mucositis | Copanlisib | 3 | 35 | 184 | 0.1827[0.1272;0.2382] | P=0.2064 |  | 1 | 4 | 142 | 0.0282 | — |
|  | Including copanlisib plus rituximab | 2 | 47 | 328 | 0.2576[0.0437;0.4715] |  |  | 2 | 3 | 328 | 0.0000[0.0000;0.0031] |  |
|  | Overall | 5 | 82 | 512 | 0.1495[0.1190;0.1801] |  |  | 3 | 7 | 470 | 0.0007[0.0000;0.0098] |  |

**Table S3.** **The incidence of adverse events of copanlisib, idelalisib, and duvelisib.**

| AEs | Copanlisib | | Idelalisb | | | | | | Duvelisib | | | | | |
| --- | --- | --- | --- | --- | --- | --- | --- | --- | --- | --- | --- | --- | --- | --- |
|  | Our meta-analysis | | Phase I(9) | | Phase I(10) | | Phase II(11) | | Phase I(12) | | Phase I(13) | | Phase II(14) | |
|  | Any grade (%) | grade ≥3 (%) | Any grade (%) | grade≥3 (%) | Any grade (%) | grade≥3 (%) | Any grade (%) | grade≥3 (%) | Any grade (%) | grade≥3 (%) | Any grade (%) | grade≥3 (%) | Any grade (%) | grade≥3 (%) |
| Neutropenia | 22.8 | 18 | 30 | 10 | — | — | 56 | 27 | 38.6 | 20 | 38.7 | 32.2 | 28.7 | 24.8 |
| Anemia | 21.9 | 5.2 | 22.5 | 2.5 | — | — | 28 | 2 | 24.8 | 14.3 | 19.4 | 12.9 | 26.4 | 14.7 |
| Decreased platelet count | 14.4 | 4.4 | 20 | 5 | 25 | 10.9 | 26 | 6 | 23.3 | 14.3 | 19.4 | 6.4 | 18.6 | 11.6 |
| Pyrexia | 26.5 | 2.6 | 27.5 | 0 | 20.3 | 3.1 | 28 | 2 | 35.2 | 1.4 | 51.6 | 3.2 | 24.8 | 0 |
| Headache | 16.6 | 0 | 15 | 0 | — | — | 10 | 1 | 18.1 | 1.9 | 25.8 | 3.2 | 15.5 | 0 |
| Decreased appetite | 12.3 | 0 | 20 | 15 | — | — | 18 | 1 | 21 | 1 | 22.6 | 0 | 14.7 | 0.8 |
| Fatigue | 32.8 | 1.9 | 25 | 2.5 | 35.9 | 3.1 | 30 | 2 | 40.5 | 8.6 | 41.9 | 0 | 27.9 | 4.7 |
| Diarrhea | 35.9 | 2.9 | 40 | 17.5 | 35.9 | 9.4 | 43 | 13 | 41.9 | 11.4 | 54.8 | 25.8 | 48.4 | 14.7 |
| Nausea | 35.8 | 0.9 | 32.5 | 5 | 25 | 1.6 | 30 | 2 | 31.9 | 3.3 | 38.7 | 6.5 | 29.5 | 1.6 |
| Vomiting | 15.1 | 0.2 | 12.5 | 0 | 12.5 | 0 | 15 | 2 | 17.6 | 1.9 | 19.4 | 3.2 | 18.6 | 3.9 |
| Constipation | 13.8 | 0 | 15 | 0 | 12.5 | 0 | — | — | 15.2 | 0.5 | — | — | 11.6 | 0 |
| Cough | 18.4 | 0.2 | 12.5 | 0 | 18.8 | 1.6 | 29 | 0 | 31.4 | 0.5 | 38.7 | 0 | 27.1 | 0 |
| Upper respiratory tract infection | 11.2 | 1.41 | 20 | 0 | 17.2 | 0 | 14 | 0 | 16.2 | 0.5 | 19.4 | 0 | — | — |
| Dyspnea | 11 | 4.5 | 10 | 0 | — | — | 18 | 3 | 19.5 | 5.3 | 22.6 | 0 | — | — |
| Pneumonia | 14 | 10.6 | 12.5 | 10 | 18.8 | 17.2 | 11 | 7 | 13.3 | 9.5 | — | — | 7.8 | 5.4 |
| ALT increased | 10.1 | 0.6 | 50 | 20 | 48.4 | 23.4 | 47 | 13 | 38.6 | 19.5 | 58.1 | 38.7 | 14 | 5.4 |
| AST increased | 2.1 | 0 | 60 | 15 | 53.1 | 20.3 | 35 | 8 | 37.6 | 15.3 |  |  | 10.1 | 3.1 |
| Hyperglycemia | 63.7 | 37.6 | 27.5 | 0 | 39.1 | 1.6 | — | — | — | — | — | — | — | — |
| Hypertension | 49.7 | 27.6 | — | — | — | — | — | — | — | — | — | — | — | — |
| Rash | 15.2 | 1.7 | 22.5 | 2.5 | 25 | 3.1 | 13 | 2 | 14.3 | 0.5 | 22.6 | 0 | 18.6 | 4.7 |
| Oral mucositis | 18.3 | 2.8 | — | — | — | — | — | — | — | — | 16.1 | 0 | — | — |

**Reference**

1. Liu W, Ping L, Xie Y, Sun Y, Du T, Niu Y, et al. A phase I pharmacokinetic study of copanlisib in Chinese patients with relapsed indolent non-Hodgkin lymphoma. Cancer chemotherapy and pharmacology. 2022;89(6):825-31.

2. Lenz G, Hawkes E, Verhoef G, Haioun C, Thye Lim S, Seog Heo D, et al. Single-agent activity of phosphatidylinositol 3-kinase inhibition with copanlisib in patients with molecularly defined relapsed or refractory diffuse large B-cell lymphoma. Leukemia. 2020;34(8):2184-97.

3. Dreyling M, Morschhauser F, Bouabdallah K, Bron D, Cunningham D, Assouline SE, et al. Phase II study of copanlisib, a PI3K inhibitor, in relapsed or refractory, indolent or aggressive lymphoma. Annals of oncology : official journal of the European Society for Medical Oncology. 2017;28(9):2169-78.

4. Dreyling M, Santoro A, Mollica L, Leppä S, Follows G, Lenz G, et al. Long-term safety and efficacy of the PI3K inhibitor copanlisib in patients with relapsed or refractory indolent lymphoma: 2-year follow-up of the CHRONOS-1 study. American journal of hematology. 2020;95(4):362-71.

5. Patnaik A, Appleman LJ, Tolcher AW, Papadopoulos KP, Beeram M, Rasco DW, et al. First-in-human phase I study of copanlisib (BAY 80-6946), an intravenous pan-class I phosphatidylinositol 3-kinase inhibitor, in patients with advanced solid tumors and non-Hodgkin's lymphomas. Annals of oncology : official journal of the European Society for Medical Oncology. 2016;27(10):1928-40.

6. Morschhauser F, Machiels JP, Salles G, Rottey S, Rule SAJ, Cunningham D, et al. On-Target Pharmacodynamic Activity of the PI3K Inhibitor Copanlisib in Paired Biopsies from Patients with Malignant Lymphoma and Advanced Solid Tumors. Molecular cancer therapeutics. 2020;19(2):468-78.

7. Matasar MJ, Capra M, Özcan M, Lv F, Li W, Yañez E, et al. Copanlisib plus rituximab versus placebo plus rituximab in patients with relapsed indolent non-Hodgkin lymphoma (CHRONOS-3): a double-blind, randomised, placebo-controlled, phase 3 trial. The Lancet Oncology. 2021;22(5):678-89.

8. Matasar MJ, Dreyling M, Leppä S, Santoro A, Pedersen M, Buvaylo V, et al. Feasibility of Combining the Phosphatidylinositol 3-Kinase Inhibitor Copanlisib With Rituximab-Based Immunochemotherapy in Patients With Relapsed Indolent B-cell Lymphoma. Clinical lymphoma, myeloma & leukemia. 2021;21(11):e886-e94.

9. Kahl BS, Spurgeon SE, Furman RR, Flinn IW, Coutre SE, Brown JR, et al. A phase 1 study of the PI3Kδ inhibitor idelalisib in patients with relapsed/refractory mantle cell lymphoma (MCL). Blood. 2014;123(22):3398-405.

10. Flinn IW, Kahl BS, Leonard JP, Furman RR, Brown JR, Byrd JC, et al. Idelalisib, a selective inhibitor of phosphatidylinositol 3-kinase-δ, as therapy for previously treated indolent non-Hodgkin lymphoma. Blood. 2014;123(22):3406-13.

11. Gopal AK, Kahl BS, de Vos S, Wagner-Johnston ND, Schuster SJ, Jurczak WJ, et al. PI3Kδ inhibition by idelalisib in patients with relapsed indolent lymphoma. The New England journal of medicine. 2014;370(11):1008-18.

12. Flinn IW, O'Brien S, Kahl B, Patel M, Oki Y, Foss FF, et al. Duvelisib, a novel oral dual inhibitor of PI3K-δ,γ, is clinically active in advanced hematologic malignancies. Blood. 2018;131(8):877-87.

13. Flinn IW, Patel M, Oki Y, Horwitz S, Foss FF, Allen K, et al. Duvelisib, an oral dual PI3K-δ, γ inhibitor, shows clinical activity in indolent non-Hodgkin lymphoma in a phase 1 study. American journal of hematology. 2018;93(11):1311-7.

14. Flinn IW, Miller CB, Ardeshna KM, Tetreault S, Assouline SE, Mayer J, et al. DYNAMO: A Phase II Study of Duvelisib (IPI-145) in Patients With Refractory Indolent Non-Hodgkin Lymphoma. Journal of clinical oncology : official journal of the American Society of Clinical Oncology. 2019;37(11):912-22.
